# Evaluation of MPEG-7-Based Audio Descriptors for Animal Voice Recognition over Wireless Acoustic Sensor Networks

**DOI:** 10.3390/s16050717

**Published:** 2016-05-18

**Authors:** Joaquín Luque, Diego F. Larios, Enrique Personal, Julio Barbancho, Carlos León

**Affiliations:** Department of Electronic Technology, University of Seville, Seville 41011, Spain; dlarios@us.es (D.F.L.); epersonal@us.es (E.P.); jbarbancho@us.es (J.B.); cleon@us.es (C.L.)

**Keywords:** sensor network, habitat monitoring, audio monitoring

## Abstract

Environmental audio monitoring is a huge area of interest for biologists all over the world. This is why some audio monitoring system have been proposed in the literature, which can be classified into two different approaches: acquirement and compression of all audio patterns in order to send them as raw data to a main server; or specific recognition systems based on audio patterns. The first approach presents the drawback of a high amount of information to be stored in a main server. Moreover, this information requires a considerable amount of effort to be analyzed. The second approach has the drawback of its lack of scalability when new patterns need to be detected. To overcome these limitations, this paper proposes an environmental Wireless Acoustic Sensor Network architecture focused on use of generic descriptors based on an MPEG-7 standard. These descriptors demonstrate it to be suitable to be used in the recognition of different patterns, allowing a high scalability. The proposed parameters have been tested to recognize different behaviors of two anuran species that live in Spanish natural parks; the *Epidalea calamita* and the *Alytes obstetricans* toads, demonstrating to have a high classification performance.

## 1. Introduction

Nowadays, there is a growing interest in studying the evolution of certain environmental parameters associated with climate change. The scientific studies of the evidence of climate change through animals is done through phenology [1]. A clear example of these effects can be observed in animal behavior [2].

One of the most common methods to evaluate animal behavior is using an acoustic study. It consists of recording and analyzing animal voices in an area of interest. These studies can help us to understand key issues in ecology, such as the health status of an animal colony. Consequently, this is an important research area in ethology.

With the purpose of acquiring animal sound patterns, ethologists have traditionally deployed audio recording systems over the natural environment where their research was being developed. However this procedure requires human presence in the area of interest at certain moments. To avoid these events, some data loggers have been proposed in the literature [3,4]. Although they reduce the monitoring effort, they still require collecting the acquired information *in-situ*. In this regard, Banerjee *et al*. [5], Dutta *et al*. [6] and Diaz *et al*. [7] proposed different remotely accessible systems in order to minimize the impact of the presence of human beings in the habitat that is being monitored.

Once the data is collected through any of the described systems, several audio processing techniques have to be developed in order to extract useful information about the animals’ behavior. This task represents a heavy workload for biologists. Therefore, automated techniques have to be considered. In this sense, some processing systems have been proposed in the literature. There are many strategies that can be followed in order to extract features from the audio records. Some examples are [8,9] where automatic audio detection and localization systems are described. Other examples are [10,11], where the authors presented an automatic acoustic recognition system for frog classification; Chunh *et al*. [12] proposes an automatic detection and recognition system for pig diseases, based on the evaluation of Mel Frequency Cepstrum Coefficients; and Kim *et al*. [13] details an automatic acoustic localization system that detect events using direct waveform comparison with acoustic patterns.

Automatic acoustic recognition systems in natural environments are not used only for animal detection. There are other applications that are based on this scheme of information extraction. This is the case of [14], which describes a detection and location system for an illegal presence in a natural park. This system is based on the calculation of the differences between the time of arrival of the sound waves emitted by multiple microphones. However, all of these *ad-hoc* solutions are specific for an application. Consequently, they cannot be scaled well to study other phenomena, such as the recognition of a new animal or a new behavior.

To overcome these limitations, this paper proposes a different approach based on the use of the MPEG-7 descriptors. MPEG-7 [15] is a multimedia content description standardized by ISO and IEC as the ISO/IEC 15938 standard. MPEG-7 was designed initially to propose a family of multimedia descriptors that allow a fast and efficient search for material that is of interest to the user.

In this paper, the MPEG-7 low level audio descriptors [16] are called primary descriptors. There are some studies that have demonstrated that these audio descriptors are suitable to be used to detect audio patterns over not well defined or variable scenarios [17], such as music identification [18], voice diseases [19], speech emotion recognition [20] or bird detection [21].

One important audio characteristic of animal voices is reverberation which can be used in animal detection and recognition. However, the basic primary descriptors do not depict these characteristics well. Due to that and taking into account applications for animal monitoring, we propose the addition of other parameters to characterize reverberations. These parameters are called secondary descriptors and are based on long-term analysis of the variations of primary descriptors.

Therefore, we propose the use of a Wireless Acoustic Sensor Network (WASN) to monitor animals’ voices. In the proposed architecture, primary descriptors are only sent through the networks, reducing the required bandwidth. Thanks to that, it is possible to use Low-Power Wireless radio communication devices, improving the network lifetime. Furthermore, the base station only requires storing primary and secondary descriptors, saving a high amount of dedicated storage space for long term environmental analysis. This information is usually enough to solve animals’ song recognition problem, as is described in Section 3.

The proposed recognition algorithm allows us to search and detect animal voices, even for animals not initially considered. In this regard, an adequate classifier must be selected and used.

The proposed distributed monitoring architecture has been mainly designed to be spread out in Spanish natural parks. This is the case of Doñana Natural Park, a park that is internationally well known for its research in the application of novel sensor technologies for environmental monitoring [22]. In this way, the proposed descriptors have been tested using a Spanish anuran audio database, obtaining promising results in the detection of common anuran species that live in these environments. This fact makes the work of the biologists easier, letting them avoid tedious tasks such as audio collection and analysis of large sets of audio patterns.

This work has been done in collaboration with the Doñana Biological Station and the National Museum of Natural Sciences in Madrid, Spain. According to our results, the proposed classifier can not only be used to distinguish an individual of Spanish species among other ones, it even detects different behaviors of the same species with accuracy. These results can be even obtained only using a small group of descriptors.

The rest of the paper is organized as follows: Section 2 and Section 3 describe the architecture of the proposed system for audio monitoring and its audio descriptors. These descriptors have been evaluated in a real case study with real anuran sounds in Section 4. Finally, Section 5 sums up the conclusions, final remarks and presents future work.

## 2. Acquisition Architecture of the Recognition System

Wireless Acoustic Sensor Networks (WASNs) represent a new paradigm for acoustic signal acquisition. They typically consist of nodes with microphones, signal processing and communication capabilities. WASNs find use in several applications, such as hearing aids, ambient intelligence, hands-free telephony, and acoustic monitoring [23].

WASN has several advantages. For example, this type of network improves the coverage area in comparison with single recording devices due to the limited operative area of a microphone. Thanks to this, this technology allows us to monitor large areas of interest, such as big natural parks like Doñana. It can also be solved using microphone arrays, but the deployment cost of a WASN is generally lower than the deployment cost of a microphone arrays.

Moreover, the redundancy of the acquired information that appears in WASNs adds extra advantages to the recognition systems. In a WASN it is possible to use pattern classifiers that consider information from multiple acoustic nodes to improve the pattern recognition and localization in environmental applications [24].

**Figure 1:**
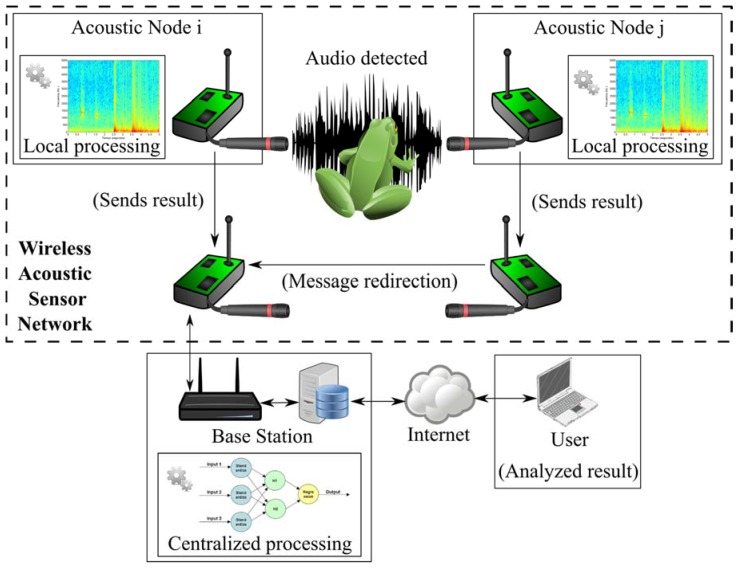
Architecture of the proposed Wireless Acoustic Sensor Network.

All these reasons inspired the use of a WASN for animals’ voice recognition in environmental monitoring scenarios. In this case, a system with the architecture depicted in Figure 1 is proposed. As it can be seen, it is based on two different devices: the Acoustical Nodes (*i.e.*, the smart measurement devices) and the Base Station, which collaborates amongst them to solve the animals’ voice recognition problem. Both types of devices are described briefly in the following subsections.

### 2.1. Wireless Acoustic Nodes

**Figure 2:**
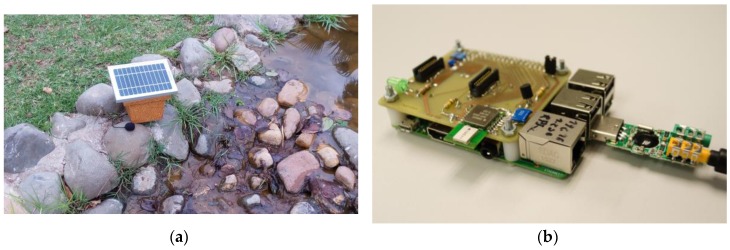
Current prototype of the acoustic sensor: (**a**) External enclosure; (**b**) Sensor board.

The proposed acoustic nodes, are based on a small low-power ARM architecture, with a radio communication module and an audio interface. This node and the enclosure for its use in environmental applications is depicted in Figure 2.

Essentially, this acoustic node uses a low-cost development board based on ARM Cortex A7 with SIMD (NEON) instruction support, which can efficiently execute audio processing. This development board is credit card sized, with low power consumption. It has 1 GB of RAM and can execute a fully functional Linux OS. The board also includes multiple I/O ports that can be used to connect external sensors, such as microphones and communication interfaces.

In this way, the proposed radio module is based on the IEEE802.15.4 standard [25]. This radio transceiver is very common in Wireless Sensor Network (WSN) nodes, following the reduction of its power consumption. Moreover, this standard is able to use the 6LoWPAN stack (an IPv6-based specification especially designed for low power consumption radios), which is also used to implement a communication layer [26]. This implementation allows multi-hop communication, which extends the basic communications amongst the nodes (in the 100–300 m range).

The use of a WASN has some drawbacks, mainly, the maximum bandwidth available and the power consumption of the radio device. This is why it is necessary to reduce, as much as possible, the amount of information sent by the radio transceiver.

Sending the entire raw audio pattern using wireless communications typically requires the use of high bandwidth radio transceivers, such as IEEE 802.11 ones. However, these radio transceivers have large power consumption. Instead of using these kinds of devices, this application proposes sending a reduced amount of data per each pattern, based on the primary descriptors described below. Thanks to this feature, we can use these low power radio devices in this application.

Dealing with the audio interface being used, we have selected a USB soundcard connected to an environmental microphone, capturing its surrounding sound. In acoustic monitoring, the type of microphone and acquisition system will be selected according to the animals under study. However, due to its limited acquisition frequencies [27], in most common cases of terrestrial observations, it is enough to use audio devices that can only sense within the human audible range. This is the case in anuran surveillance, with voices in the 20–20,000 Hz range [28].

If only one microphone is used in an environmental monitoring application, it needs to be highly sensitive. It needs to be very linear and it requires a complex calibration [29]. This is why these microphones are expensive (over $200). Furthermore, we have to consider the cost of its protection to work in harsh scenarios.

**Figure 3:**
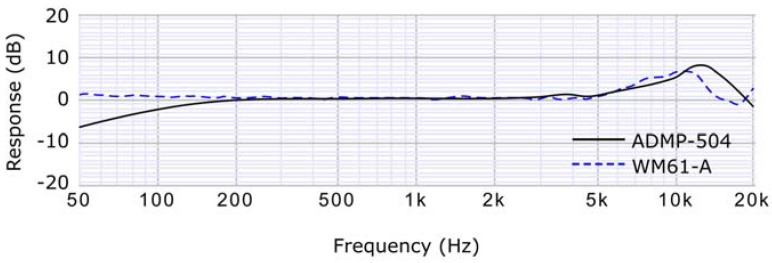
Frequency response of different microphones.

Fortunately, this restriction is relaxed if an array or a network of different microphones is used, due to the redundancy of the information that appears in different nodes. Thanks to that, it is possible to use low-cost electret microphones [30] in WASNs. Figure 3 depicts the frequency response of some of these low cost (less than $20) microphones. As it can be seen, even being cheaper, they have a good frequency response in the range required for our application.

### 2.2. Base Station

The base station (BS), also called the information service, is a PC-based device with two communication modules: one to acquire information from the wireless acoustic sensor network, and another to allow researchers to obtain the gathered information. Therefore, this device acts typically as a database, where the nodes save the information, and the user can retrieve it as needed.

However, in the proposed animal voice recognition system, the required information is not the stored one. As described in the processing architecture, we use a classifier in the BS to retrieve the audio voice recognition service. It increases the computational requirements of the base station, but allows us to detect voices of animals not considered at the moment of the deployment.

## 3. Processing Architecture of the Recognition System

**Figure 4:**
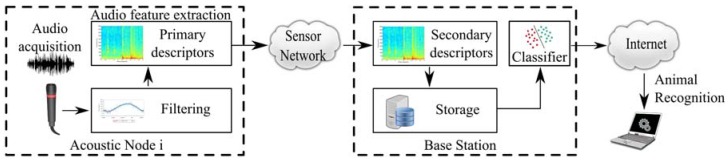
Processing architecture of the system.

The processing architecture of the animal voice detection system is depicted in Figure 4. In this implementation, audio patterns are acquired from the monitoring area by the acoustic sensors and processed locally to obtain primary descriptors in wireless acoustic nodes.

After that, these primary descriptors are sent to the BS through the wireless radio transceiver of the nodes. Then, when the BS receives this data, secondary parameters are obtained. Only primary and secondary descriptors are stored in the database, where it can be used by a classifier for animal voice detection. The next subsections describe briefly the main blocks of the processing architecture:

### 3.1. Filtering

The main objective of the filtering stage consists of reducing the amount of noise in the patterns. This goal is obtained using a band-pass filter that eliminates frequencies of the acquired audio that do not contain relevant information. This filter can be adjusted for each application. For most animal voice analysis, in the human audible range, a 0.3–10 kHz band-pass is a good tradeoff between noise elimination and information loss.

### 3.2. Primary Descriptors

This subsystem is responsible for obtaining the primary descriptors. These descriptors receive the name of Low Level Descriptors (*LLDs*) and they are defined in MPEG-7 specification.

According to this specification, *LLDs* can be obtained from the audio spectrogram. It consists of a time–frequency analysis based on obtaining the amplitude (in dB) of the frequency response of each frame. In this case, the specifications recommend using a temporal window of 10 ms as a frame.

**Figure 5:**
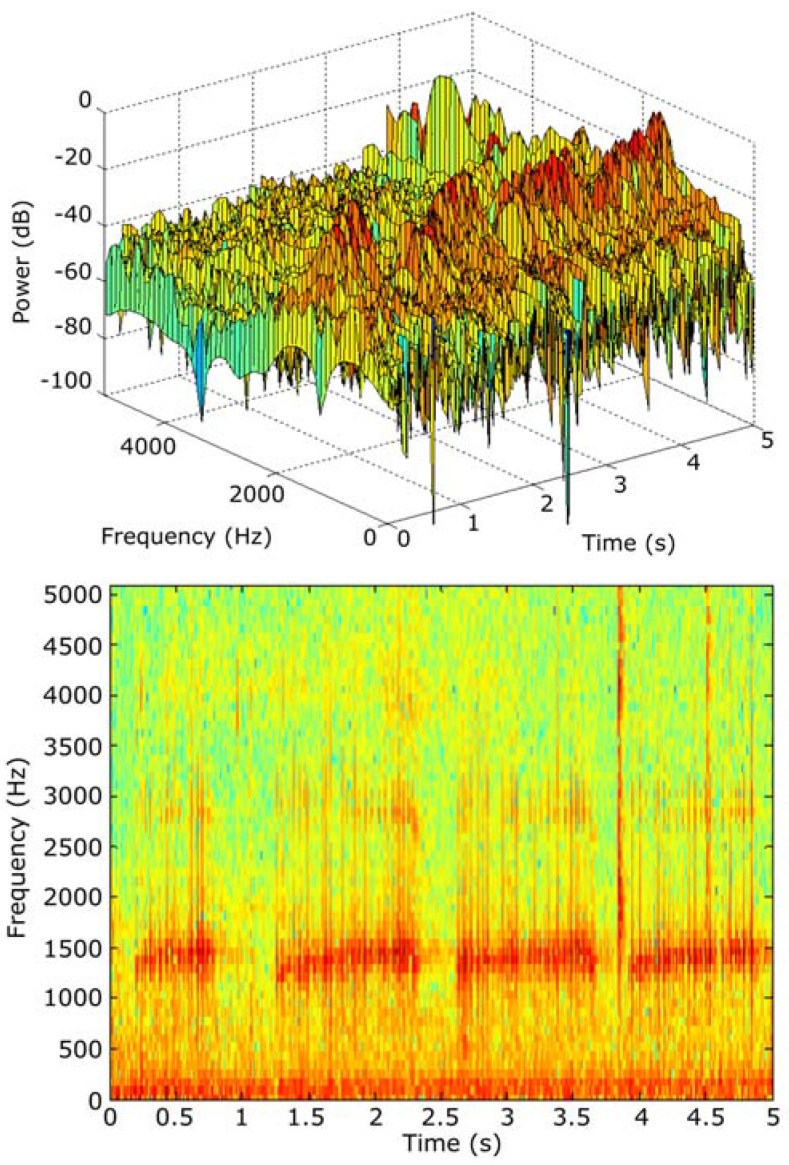
Audio spectrogram example.

As shown in Figure 5, the spectrograms are commonly depicted as an X-Y level graph, where X is the time, Y the frequency and the darkness of the image represents the relative amplitude of the power of the signal.

This spectrogram can be used by a trained user to distinguish animal audio patterns (in a human manual processing step). Nevertheless, *LLDs* can be used by an automatic system (without human intervention) to obtain similar conclusions. In our research, 18 primary descriptors have been considered. The cost associated with sending the information expressed with these descriptors represents, in the worst case (using single precision floating point), a data payload of only 72 bytes each 10 ms, instead of the 882 bytes per 10 ms required to send a standard 44.1 kHz raw audio pattern.

Therefore, this way of representing the information provides a huge data bandwidth reduction, especially if we consider that these parameters can be expressed using only 2 bytes, with a fixed point representation. This feature makes this kind of representation suitable for being used in low power radio modules with restricted bandwidth, as the ones used in typical WSN nodes (IEEE 801.15.4) [31]. *LLDs* characterize spectrograms with the following parameters:

#### 3.2.1. Frame Power

This parameter, expressed in dB, is obtained as the maximum energy of the frame, with respect to its minimum. Two different frame power indicators are defined in MPEG-7 specification:

- Relevant Power (PR): The relevant spectral power of the frame, defined in a frequency range. It is usually used normalized to the maximum power of the sound. By default, a 0.5–5 kHz band is used, due to the fact that most useful audio information for the human ear is in this range.
- Total Power (Pt): The normalized total spectral power, considering all the spectral dynamic ranges of the frame.

If the previous filter of the architecture is well fitted, both indicators may offer similar results.

#### 3.2.2. Frame Power Centroid ($C_{P}$)

In a particular frame, the power centroid briefly represents the shape of the frequency spectrum. The centroid offers information about the frequencies where most of the energy of the frame is distributed. It can be obtained as shown in Equation (1):

(1) $$C_{P}=\frac{\sum_{i}\left[f_{i}\cdot P_{i}\right]}{\sum_{i}P_{i}}$$

where $f_{i}$ is the frequency of the spectrogram, and $P_{i}$ the acoustic power of the associated frequency.

#### 3.2.3. Spectral Dispersion ($D_{E}$)

It represents approximately the shape of the frequency spectrum of a frame. It can be obtained as shown in Equation (2):

(2) $$D_{E}=\sqrt{\frac{\sum_{i}\left[{\left(f_{i}-C_{p}\right)}^{2}\cdot P_{i}\right]}{\sum_{i}P_{i}}}$$

#### 3.2.4. Spectrum Flatness ($P_{l}$)

It represents the deviation of the audio spectrum in relation to a flat spectrum (*i.e.*, a white noise). It can be obtained with Equation (3):

(3) $$P_{l}=\frac{\sqrt{\prod_{i}P_{i}}}{\frac{1}{n}\sum_{i}P_{i}}$$

#### 3.2.5. Harmonicity Ratio ($R_{a}$)

$R_{a}$ is the ratio of harmonic power to total power. According to MPEG-7 specifications, it can be obtained from the autocorrelation of the audio signal s(n) into the frame, as expressed in Equation (4):

(4) $$R_{a}=\underset{k}{\max}\left(\frac{\sum_{j}s\left(j\right)\cdot \left(j-k\right)}{\sqrt{\sum_{j}s{\left(j\right)}^{2}\cdot \sum_{j}s{\left(j-k\right)}^{2}}}\right)$$

where k is the length of the frame.

#### 3.2.6. Fundamental Frequency (Pitch)

The fundamental frequency (or sound pitch) is the frequency that best describes the periodicity of a signal. It can be obtained with the Linear Prediction Coding (*LPC*) algorithm [32]. *LPC*, models the spectral power as sum of the polynomial A(z) defined by Equation (5):

(5) $$A\left(z\right)=1-\sum_{k=1}^{p}\left[a_{k}\cdot z^{-k}\right]$$

where the polynomial degree p represents the model precision. The coefficients$\;a_{k}$ represent the formants of the audio signal, *i.e.*, the spectral maximum produces by the audio resonances. According to [32], these coefficients can be obtained reducing the error between the polynomial prediction and the spectral power of the audio frame using a minimum least squares estimation.

For the sound s(n) the error function ε(n) is obtained according to Equation (6):

(6) $$\varepsilon \left(n\right)=s\left(n\right)-\sum_{k=1}^{p}a_{k}s\left(n-k\right)$$

For ε(n), the autocorrelation function is obtained by Equation (7):

(7) $$\varphi \left(k\right)=\sum_{m=-\infty }^{+\infty }\varepsilon \left(m\right)\;\varepsilon \left(m+k\right)$$

Using this information, the Pitch can be defined as the first maximum of the φ(k) autocorrelation function.

Due to the execution complexity of *LPC* a tradeoff between time of computation and accuracy is necessary. According to our tests, a polynomial of size 30 is enough to obtain Pitch estimations with good accuracy.

#### 3.2.7. Upper Limit of Harmonicity ($F_{la}$)

As defined in Section 5.3.13.3.1 of the MPEG standard [33], this is the frequency beyond which the spectrum cannot be considered harmonic. It can be obtained using the comb filter of Equation (8):

(8) $$c\left(j\right)=s\left(j\right)-\left(\sum_{j=m}^{m+n-1}\left[s\left(j\right)\cdot s\left(j-K\right)\right]/\sum_{j=m}^{m+n-1}\left[s{\left(j-K\right)}^{2}\right]\right)\cdot s\left(j-K\right)$$

where s(n) is the audio signal and K is the delay related to $R_{a}$ (the Harmonicity Ratio).

Considering P(f) and $P^{\prime }\left(f\right)$ as the spectral power of s(n) and the filtered signal c(j), the ratio for each frequency is defined as Equation (9):

(9) $$\alpha \left(f_{\text{lim}}\right)=\sum_{f=f_{\text{lim}}}^{f_{max}}P^{\prime }\left(f\right)/\sum_{f=f_{\text{lim}}}^{f_{max}}P\left(f\right)$$

Using it, $F_{la}$ can be obtained as the maximum frequency where $\alpha \left(f_{\text{lim}}\right)$ is higher than a threshold, 0.5 in this case, as is commonly used.

#### 3.2.8. Harmonic Peaks

The harmonic peaks are the n highest peaks of the spectrum located around the multiple of the fundamental frequency of the signal. The terms that surround the fundamental frequency are used in order to take into account the slight variations of harmonicity of some sounds.

MPEG-7 describes harmonic peaks that we propose to form the following descriptors:

- Frequency of the harmonic peaks (FrFi).
- Bandwidth of the harmonic peaks (AbFi).

Both parameters can be obtained using the aforementioned *LPC* algorithm. In most practical scenarios, it is usual to consider the three first harmonic peaks to characterize the frame.

**Figure 6:**
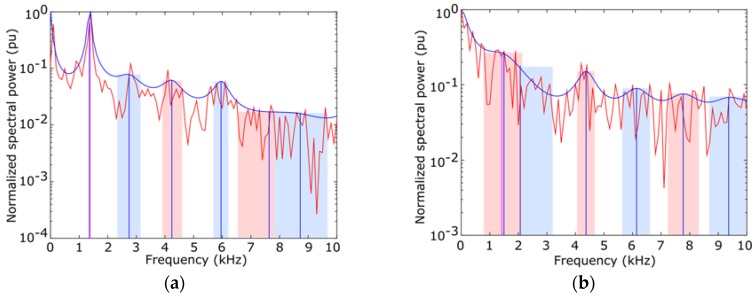
Example of results obtained with LPC analysis. (**a**) t=0.80 $f_{f}=\left[36\;,\;810\;,\;682\right]\;Hz$; (**b**) $t=2.00\;f_{f}=\left[1316,\;2191,\;647\right]\;Hz$ .

Figure 6 depicts the results of an *LPC* analysis of two different frames. The red shape is the original audio spectrum and the blue shape represents the *LPC* algorithms. Blue and red bands are the bandwidth of the harmonic peaks, whose frequency is depicted as a vertical blue line.

#### 3.2.9. Harmonic Centroid ($C_{A}$)

It is a weighted average that selects a frequency that represents the harmonic peaks of the frame. It can be obtained with Equation (10):

(10) $$C_{H}=\frac{\sum_{i=1}^{n_{f}}\left[f_{i}\cdot v_{i}\right]}{\sum_{i=1}^{n_{f}}v_{i}}$$

where $f_{i}$ is the ith frequency of the harmonic peak and $v_{i}$ its peak value.

#### 3.2.10. Harmonic Spectral Deviation ($D_{eA}$)

It is computed as the deviation between harmonic peaks and the signal spectrum of the frame. It can be obtained with Equation (11):

(11) $$D_{eA}=\underset{k}{\max}\left(\frac{\sum_{i=1}^{n_{f}}\left|\log\left(v_{i}\right)-\log\left(\frac{1}{3}\cdot \sum_{k=-1}^{1}\left[v_{i}+k\right]\right)\right|}{\sum_{i=1}^{n_{f}}\log\left(v_{i}\right)}\right)$$

#### 3.2.11. Harmonic Spectral Spread ($D_{iA}$)

It is computed as the typical deviation between harmonics peaks and the harmonic centroid. It can be obtained with Equation (12):

(12) $$D_{iA}=\frac{1}{C_{a}}\cdot \sqrt{\frac{\sum_{i=1}^{n_{f}}\left[v_{i}{}^{2}\cdot {\left(f_{i}-C_{a}\right)}^{2}\right]}{\sum_{i=1}^{n_{f}}v_{i}{}^{2}}}$$

#### 3.2.12. Harmonic Spectral Variation ($V_{A}$)

It is obtained as a normalized correlation between the harmonic peaks of two adjacent frames. It can be obtained with Equation (13):

(13) $$V_{A}=1-\frac{\sum_{i=1}^{n_{f}}\left[v_{i,j}\cdot v_{i,j-1}\right]}{\sum_{i=1}^{n_{f}}\sqrt{v_{i,j}{}^{2}}\cdot \sum_{i=1}^{n_{f}}\sqrt{v_{i,j-1}{}^{2}}}$$

where $v_{i,j}$ is the amplitude of the *i*th harmonic of the frame j.

### 3.3. Secondary Descriptors

One shortcoming in the use of MPEG-7 *LLDs* for animal voice detection is that they do not consider reverberation effects. This is due to the reduced size of the frame (only 10 ms), which is smaller than the duration of these effects. However, many animal characteristic acoustic patterns have an important reverberation component, which can be used to distinguish between species.

To describe these effects we propose the use of secondary descriptors. These descriptors are obtained with a methodology based on the same methodology used with the primary descriptors. The dispersion between longer time frames is analyzed. These time frames are called micro-segments and have a duration of 100 ms (*i.e.*, 10 frames). This time is chosen considering that, audio delays higher than 100 ms are identified by human being as echo, instead of a reverberation.

We define these secondary descriptors as the interquartile range of each one of the primary parameters, in a 100 ms micro-segment.

According this procedure, we obtained the next secondary parameters:

- Dispersion of relevant Power (PR~).
- Dispersion of total Power (Pt~).
- Dispersion of centroid of frame power (CP~).
- Dispersion of spectral dispersion (DE~).
- Dispersion of spectrum flatness (Pl~).
- Dispersion of harmonicity ratio (Ra~).
- Dispersion of fundamental frequency (Pitch~).
- Dispersion of upper limit of harmonicity (Fla~).
- Dispersion of Frequency of the harmonic peaks (FrFi~).
- Dispersion of Bandwidth of the harmonic peaks (AbFi~).
- Dispersion of harmonic centroid (CA~).
- Dispersion of harmonic spectral deviation (DeA~).
- Dispersion of harmonic spectral spread (DiA~).
- Dispersion of harmonic spectral variation (VA~).

The set of secondary descriptors does not need to be computed (and sent) by the acquisition system. Due to the fact that they are obtained based on variations of primary descriptors, they can be obtained by the BS before storing the information in the storage server.

**Figure 7:**
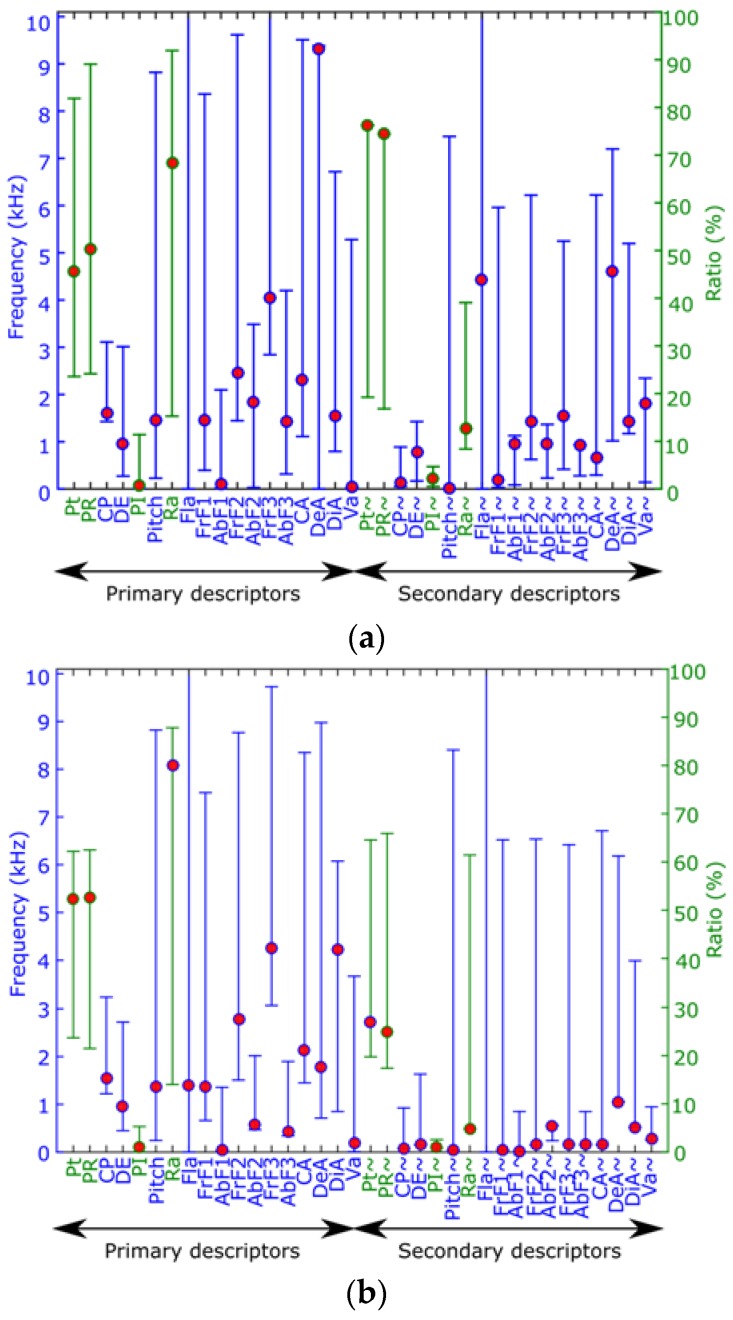
Examples of primary and secondary descriptors for two different frames. (**a**) *Epidalea calamita* (0501) *t* = 0.5; (**b**) *Alytes obstetricans* (1325) *t* = 0.8.

Figure 7 depicts examples with the values of all proposed parameters in two different samples: an *Epidalea calamita* (natterjack toad) and an *Alytes obstetricans* (common midwife toad) one. In this figure, the red circles represent the current value of each parameter in a particular frame, while the vertical line indicates its range during a full segment of the audio file.

On the other hand, the color of the axis indicates the units of the parameters. As it can be seen, red ones are defined in Hertz, while green ones are in percentages.

This example graphically shows that the proposed parameters have very different range and values for different audio samples. Therefore, they can be effectively used by an automatic classification system in order to distinguish different patterns.

### 3.4. Data Storage

This subsystem is dedicated to storing the primary and secondary descriptors. This information must be tagged with a timestamp, and can be stored in a regular SQL database.

### 3.5. Classifier

This subsystem plays an important role in the whole system. It is responsible for the implementation of the classification method. Its main goal is to classify the audio patterns (*i.e.*, its descriptors) into one of the proposed classes. In this sense, hundreds of supervised classifiers have been proposed in the literature. The selection for the best method is a decision that has to be done considering the characteristics of the problem to solve [34]. However, in our real case analysis, even simple classifiers offer good class separations using the aforementioned descriptors.

## 4. A Case Study: Anuran classification system

**Figure 8:**
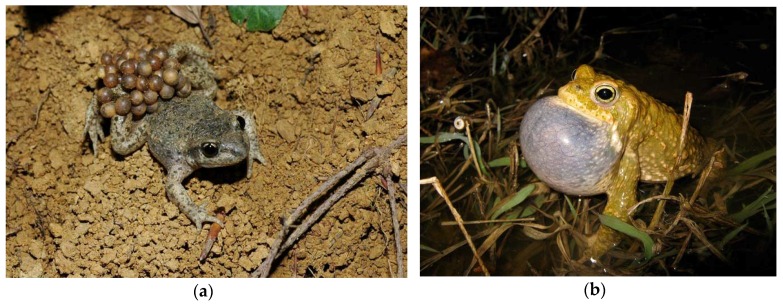
Two specific anuran species: (**a**) *Alytes obstetricans* toad, carrying egg strings [35]; (**b**) *Epidalea calamita* toad [36]. Photographs by Ursina Tobler.

The proposed animal audio detection descriptors have been evaluated considering the different behavior of two anuran species: *Alytes obstetricans* (Figure 8a) and *Epidalea calamita* (Figure 8b).

Ethology studies focused on the anuran species, such as the described ones, are especially interesting for the biologist. The reason for this interest is based on the fact that in some amphibian species, climate change and UV radiation have important effects on their population (largely determined by their habitat) [1,37]. More concretely, an interesting phenomenon can be observed in anurans (frogs and toads). These kinds of amphibians present peaks in the timing of reproductive calling (songs), depending on each species and temperature evolution [38]. This is why an anuran classifier is used as a case study of the proposed descriptors.

### 4.1. Sound Database Information

In order to analyze the performance of the proposed descriptors, this research has based its evaluation on a relevant database of anuran songs. This database is composed by a set of audio samples of *Alytes obstetricans* and *Epidalea calamita* provided by “*Fonozoo: Fonoteca Zoológica del Museo Nacional de Ciencias Naturales*”, a Spanish phonotheque based on recordings of animal sounds [39]. This organization provided a scientific collection of animal sounds. Fonozoo was created with the aim of supporting the study of acoustic communication in animals.

The collection includes more than 45,000 sound recordings belonging to more than 12,000 different species, all of which makes Fonozoo a valuable tool in the systematic study of animal behavior. The main goal of using a public audio database is that these files can be used by other researchers all over the world for comparative performance analysis. In the case of the studied anurans, the collection is composed of 63 audio samples, according to the characteristics described in Table 1.

This collection of anuran audio files offers a total of 6053 s of recorded sounds, with a mean duration of 96 s and a median of 53 s. Therefore, our test collection has 605,300 frames, where each frame has a total amount of 36 associated values that are its primary and secondary descriptors.

As can be seen, the number of chorus vocalization samples of *Epidalea calamita* toads is not enough to train a system. Moreover, it sounds similar to the standard vocalization. Due to this, both cases are going to be considered as the same classification category.

In the case of the *Alytes obstetricans* toads, there are not enough distress call vocalization samples to train a system. Due to this, in the test of the proposed descriptor, we have considered the next number of samples by categories:

- Type 1: 23 samples of *Epidalea calamita* toad with standard or chorus vocalization.
- Type 2: 10 samples of *Epidalea calamita* toad with oviposit vocalization.
- Type 3: 30 samples of *Alytes obstetricans* toad with standard or distress call vocalization.

Due to the fact that the samples have been obtained from environmental scenarios, not only animal vocalization is recorded. In fact, there is a high percentage of background sound, composed of wind, rain, traffic, human voices, and so forth. Therefore, there are intervals of time in each sample that do not correspond with animal vocalization, and which need to be detected and rejected by the classification system. With this purpose, a fourth type of audio has been considered, that corresponds with noise, *i.e.*, sounds in which we are not interested in.

### 4.2. Classification Results

Supervised classifier techniques require the use of sound models for each class. These models are required in the training and adjusting phase of the classifier. In this case, two files have been chosen as a pattern from both *Epidalea calamita* and *Alytes obstetricans* toads with standard vocalization, and one file for *Epidalea calamita* toad with oviposit vocalization. These models have been selected empirically, looking for files in which the sound is easily distinguishable. In this case, files “0501” and “0707” from Fonozoo collection are chosen as type 1 pattern, “0503” as type 2, and “1325” and “1330” as type 3.

**Figure 9:**
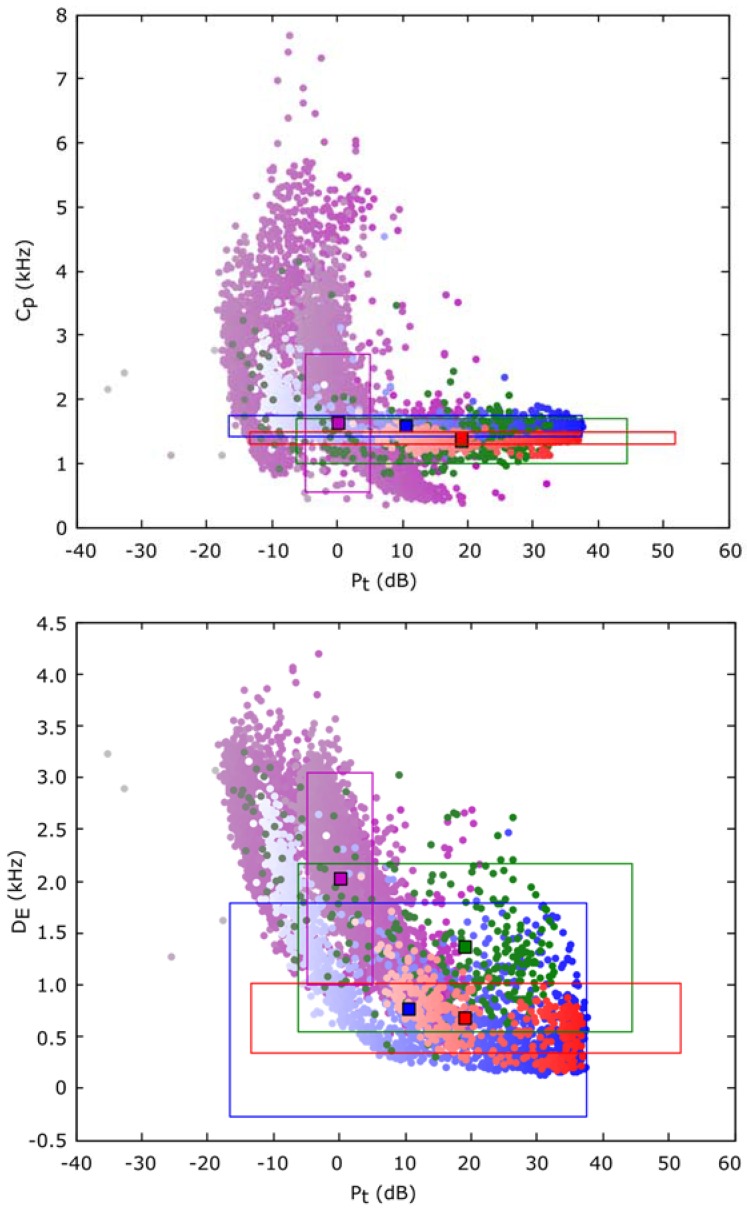
Point cloud of $C_{p}$ and $D_{E}$ descriptors *versus* its power in patterns.

Once the classification criterion has been selected, it is necessary to evaluate its value. In this sense, a classification can only offer good accuracy if there exists a surface in the input parameters (*i.e.*, the descriptors) space, where the different classes can be isolated completely or with a reduced error. This feature can be observed in Figure 9, where the value of each parameter (centroid and dispersion *versus* power in this example) for each frame is summarized. A color code has been chosen to indicate its class type. Blue for type 1 (*Epidalea calamita* toad with standard or chorus vocalization), green for type 2 (*Epidalea calamita* toad with oviposit vocalization), red for type 3 (*Alytes obstetricans* toad with standard or distress call vocalization) and purple for type 4 (noise).

It is important to take into account that although there are 36 parameters (18 primary and 18 secondary), it is not necessary to impose the use of all of them in the classifier task. Furthermore, in order to improve the response time, it is better to choose a representative subgroup of descriptors as inputs for the classifier in function of the voice animal to be recognized. In this case, we empirically chose the next descriptors for the recognition of the different voices of the *Epidalea calamita* and the *Alytes obstetricans* toads:

- Harmonicity ratio (Ra).
- Bandwidth of the first harmonic peaks (AbF1).
- Dispersion of total Power (Pt~).

As it can be seen, only three descriptors are used, two primary and one secondary. Under these conditions, the following classifiers have been tested:

#### 4.2.1. Minimum Distance Classifier

The minimum distance classifier [40] is one of the simplest, classic classification algorithms. In spite of its simplicity, it offers good accuracy in many situations. Some animal voice classification systems, such as [11] for anuran species, are based on this classifier.

The classification is based on obtaining the distance $d_{ij}$ between the input vector to classify i with each one of the representative class j. This distance can be obtained with the Euclidean distance, according to Equation (14):

(14) $$d_{ij}=\sqrt{\sum_{k=1}^{P}{\left[x_{ik}-x_{jk}\right]}^{2}}$$

where $x_{ik}$ is the value of the k-th parameter in the i-th frame.

According to it, the input can be classified as the class that minimizes the distance with it. However, the units of each parameter of the proposed system are clearly different. Therefore, a parameter normalization is needed. In this case, we propose the use a of spread range of $R_{k}$ for each parameter, which can be obtained as Equation (15) describes:

(15) $$R_{k}=\underset{i}{\max}\left(x_{ik}\right)-\underset{i}{\min}\left(x_{ik}\right)$$

Therefore, the normalized distance $nd_{ij}$ can be obtained according to Equation (16):

(16) $$nd_{ij}=\sqrt{\sum_{k=1}^{P}{\left[\frac{x_{ik}-x_{jk}}{R_{k}}\right]}^{2}}$$

If this classifier is applied to each frame of an audio pattern, the final classification of the audio sample can be obtained as the most likely class to appear in the pattern. Moreover, it can be used as a qualify indicator Q. If we consider $n_{a}$ as the number of frames correctly classified, and $n_{j}$ the total number of frames, Q can be obtained as expressed by Equation (17):

(17) $$Q=\frac{n_{A}}{\sum_{j}n_{j}}$$

**Figure 10:**
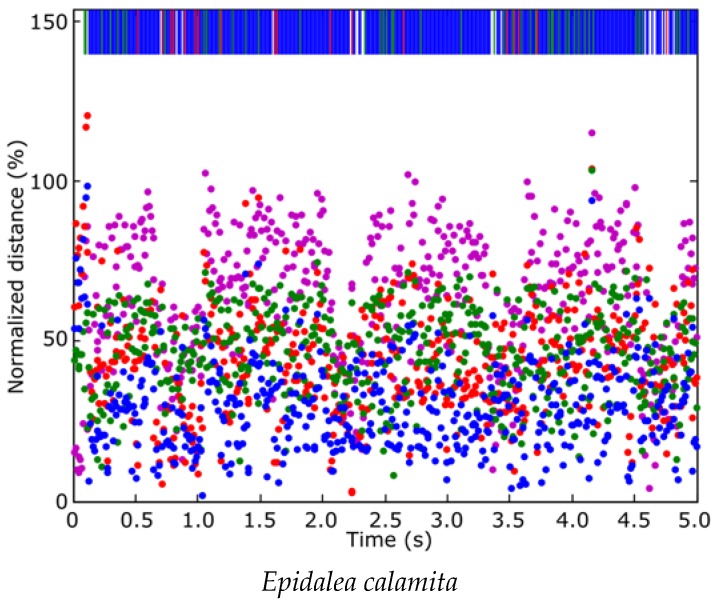
Frame classification of “0560” sample (*Epidalea calamita* in standard vocalization).

Figure 10 depicts the classification distance from each frame of the “0560” sample to the four classes. Blue indicates type 1 (*Epidalea calamita* toad with standard or chorus vocalization), green for type 2 (*Epidalea calamita* toad with oviposit vocalization), red for type 3 (*Alytes obstetricans* toad with standard or chorus vocalization) and purple for type 4 (noise). The top band of the figure indicates the minimum distance class. As can be seen, most patterns are classified correctly. In this example the quality indicator is 84.36%.

**Figure 11:**
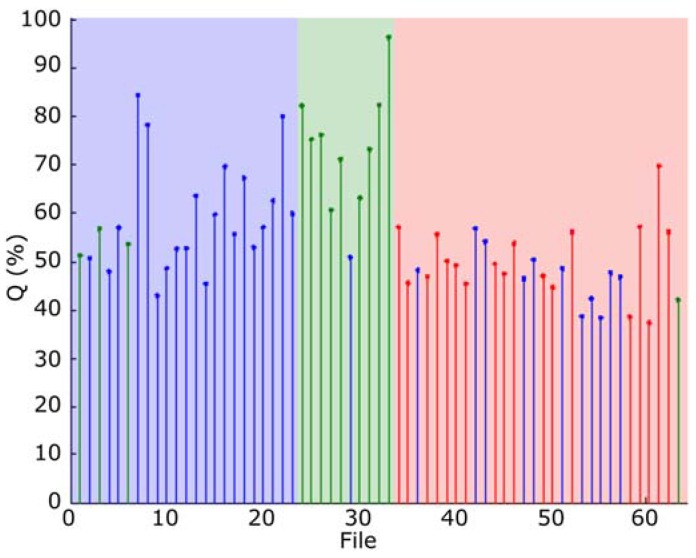
Classification result of all the samples of the audio collection.

The classification results, obtained with all the frames, are depicted in Figure 11, where the quality indicator Q, has been represented. In this figure the blue area indicates type 1 (*Epidalea calamita* toad with standard or chorus vocalization), green area type 2 (*Epidalea calamita* toad with oviposit vocalization) and red type 3 (*Alytes obstetricans* toad with standard or chorus vocalization). Vertical colored lines indicate the estimated classification. Therefore, a pattern corresponding to a file is correctly classified if its line color matches the band color.

As it can be seen, correctly classified patterns have high Q values. Then, Q can be used to determine a confidence degree in the classification result.

**Figure 12:**
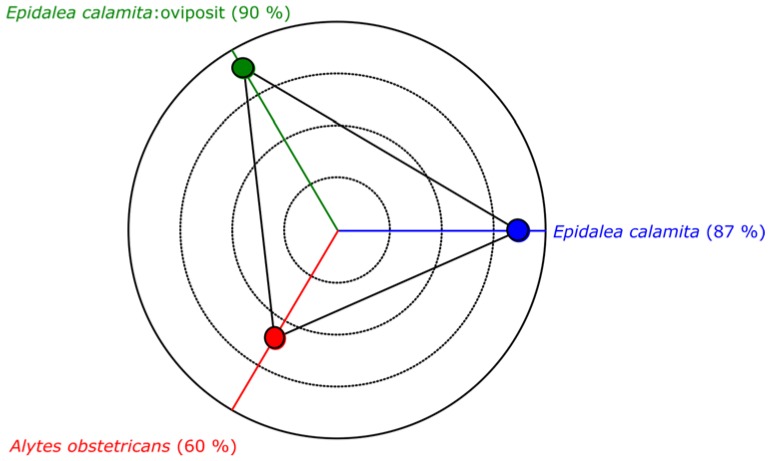
Performance of minimum distance classifier.

Figure 12 depicts the performance of the minimum distance classifier, according to the results obtained with the audio collection. As it can be seen, even a simple classifier as the minimum distance offers an acceptable classification result.

#### 4.2.2. Modified Maximum Likelihood Classification

Maximum likelihood classification [41] is another classic supervised classification algorithm which has been widely used in the literature. This classification algorithm is divided into two stages: training and execution.

The training stage is executed only once, as a previous step to classification. In this stage the statistical information to execute the classification algorithm is obtained. In the case of the maximum likelihood classification consists of generating several probability density functions $f_{\theta }\left(x_{k}\right)$ for each one of the θ classes to be detected. $f_{\theta }\left(x_{k}\right)$ is a multivariable function that is going to be used to obtain a numeric probability for each class and frame to analyze in the execution stage.

**Figure 13:**
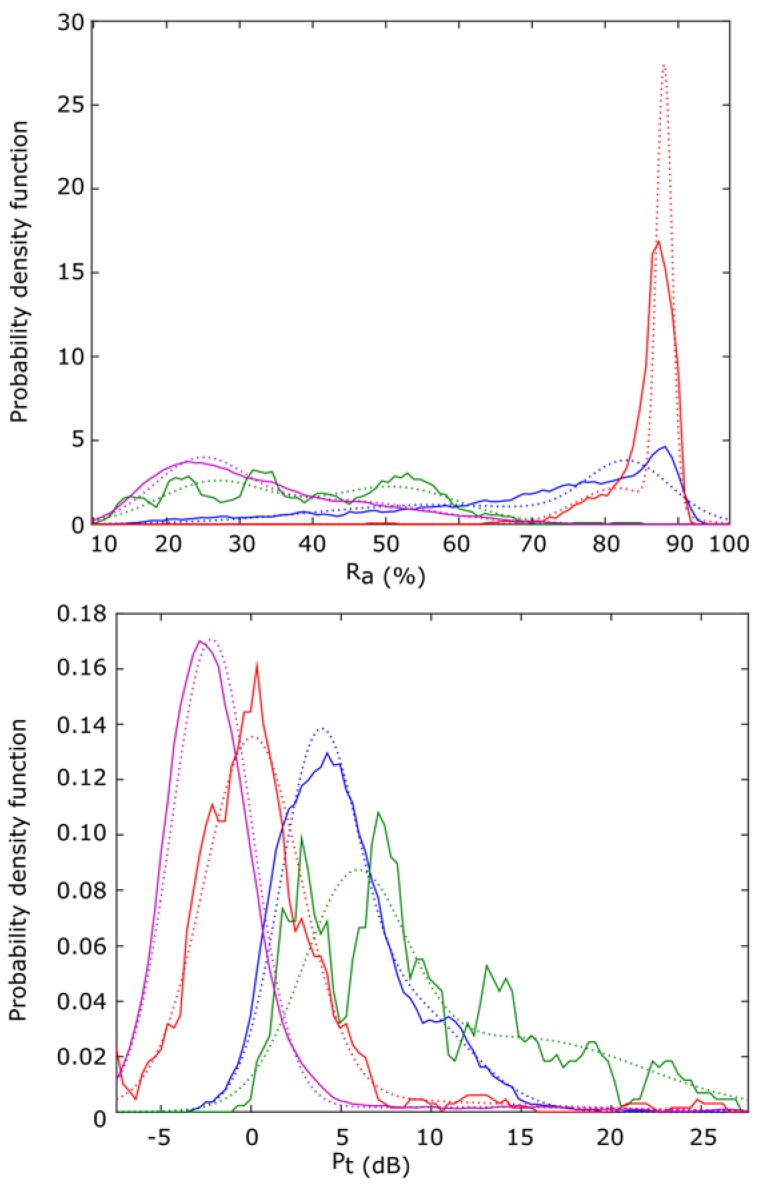

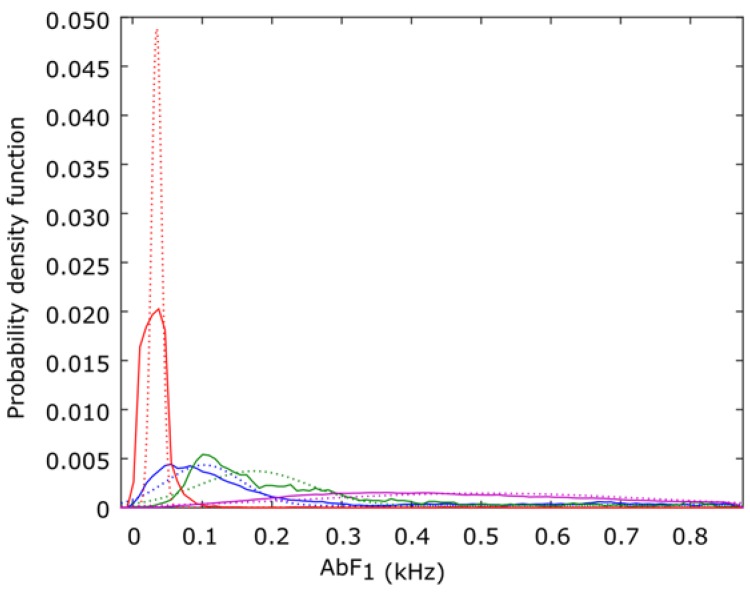
Probability density functions for each descriptor.

In this case, we propose using a Gaussian mixture distribution (GMD) [42] of two Gaussians as $f_{\theta }\left(x_{k}\right)$, obtained from the audio models. It is depicted in Figure 13, where the blue line indicates class type 1 (*Epidalea calamita* toad with standard or chorus vocalization); green type 2 (*Epidalea calamita* toad with oviposit vocalization); red type 3 (*Alytes obstetricans* toad with standard or distress call vocalization) and purple type 4 (noise). Continuous lines depict raw data from the models, while dotted lines are the GMD ones.

In execution time, maximum likelihood classification uses these $f_{\theta }\left(x_{k}\right)$ functions to obtain the probability of each frame to be one of the proposed θ classes. To do that, this algorithm uses the likelihood of the descriptors of a frame, in relation to the classes to recognize ℒ(θ|x). It can be obtained using the Expression (18):

(18) $$ℒ\left(\theta |x\right)=P\left(x|\theta \right)=f_{\theta }\left(x\right)$$

This expression give us a numeric likelihood of each class for all the frames in a sample to analyze. But to reduce the calculus complexity in maximum likelihood classification, the log of the likelihood V(θ|x) is commonly used. It can be done with the Equation (19):

(19) $$V\left(\theta |x\right)=log\left[ℒ\left(\theta |x\right)\right]=log\left[P\left(x|\theta \right)\right]=log\left[f_{\theta }\left(x\right)\right]$$

In the modified maximum likelihood algorithm, we propose using to classify the differential likelihood $D_{ij}$ instead of V(θ|x). $D_{ij}$ of a frame i is the difference of the likelihood of belonging to that class j (type 1 to 3), rather than the likelihood it being noise r (type 4). It can be obtained with the Equation (20):

(20) $$D_{ij}\equiv V\left(\theta_{j}|x_{i}\right)-V\left(\theta_{r}|x_{i}\right)=log\left[f_{\theta j}\left(x_{i}\right)\right]-log\left[f_{\theta r}\left(x_{i}\right)\right]$$

**Figure 14:**
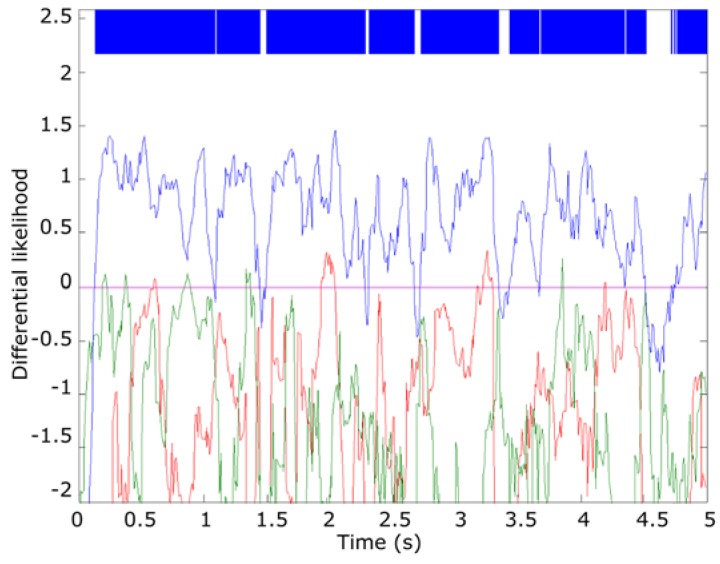
Result of the smoothed differential likelihood of “0560” sample.

In the proposed algorithm, the temporal evolution of $D_{ij}$ of a sample to analyze is smoothed out using a size 10 moving average filter to reduce spurious changes in $D_{ij}$ evolution. For classification purposes, only the maximum of $D_{ij}$ for each frame is considered. Figure 14 summarizes the results of this step for the “0560” file, an *Epidalea calamita* example. As it can be seen, in this step most of the frames are yet to be correctly classified.

**Figure 15:**
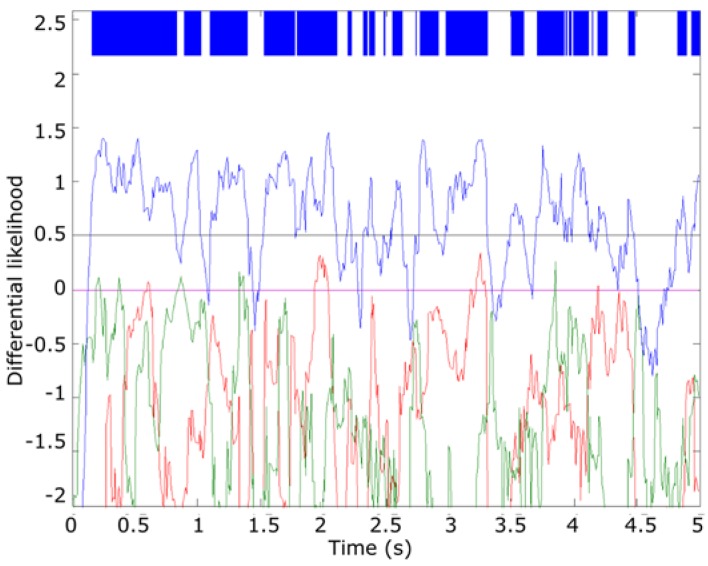
Result of the threshold application.

To increase the detection accuracy, only filtered $D_{ij}$ higher than a threshold β are considered for classification proposes. β is adjusted empirically at 30% of the relative maximum of the sample (approx. 0.5 for this example). Figure 15 summarizes the result of this step for the “0560” file. As it can be seen, in this case all the frames higher than the threshold are correctly classified.

To estimate the class θ of the audio sample, a qualifier indicator $Q_{\theta }$ is defined, according to Equation (21):

(21) $$Q_{\theta }=\frac{A_{\theta }}{\sum_{j}A_{j}}$$

$Q_{\theta }$ does not use only the maximum of the smoothed $D_{ij}$ count, but the area $A_{\theta }$ of the curve higher than the threshold β, According to our analysis, it increases the classification accuracy. Considering this information, the final estimation of the class of the sample is the one with the maximum $Q_{m}$ indicator, according to Equation (22):

(22) $$Q=\underset{j}{\max}\left(Q_{j}\right)$$

As summarized, the proposed modified maximum likelihood classificatory is based on a $log\left[f_{\theta }\left(x\right)\right]$ functions obtained from the model of the defined four classes θ. Using this information, the algorithm consist of the execution of the next steps:

- Obtain $D_{ij}=log\left[f_{\theta j}\left(x\right)\right]-log\left[f_{\theta r}\left(x\right)\right]$ for the three possible classes of sound.
- Filter $D_{ij}$ with a size 10 moving average filter.
- Select the class for each frame, as the maximun $D_{ij}$ for each class j, if its value is higher than the threshold β. Otherwise the frames are discarded from the classification.
- Obtain the final class estimation as the class with maximum area over the threshold β.

**Figure 16:**
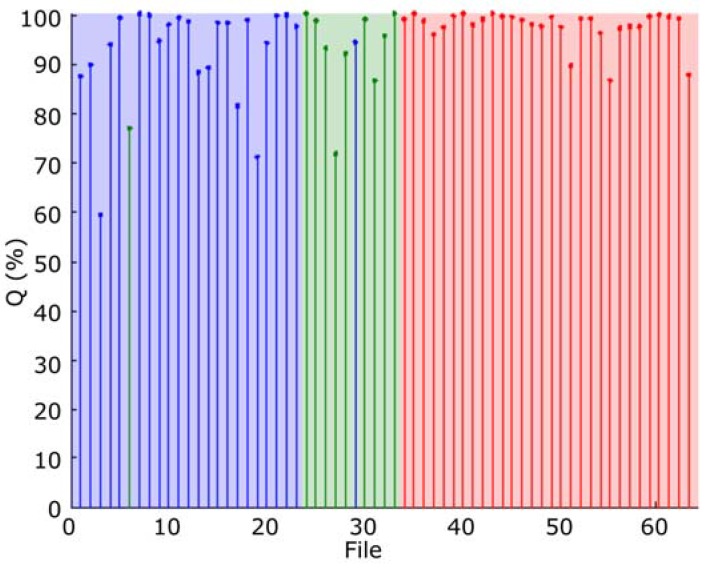
Classification result of all the samples of the audio collection.

The classification results obtained with all the frames, using this classification method is depicted in Figure 16. In this figure, the blue area indicates type 1 (*Epidalea calamita* toad with standard or chorus vocalization), green area type 2 (*Epidalea calamita* toad with oviposit vocalization) and red type 3 (*Alytes obstetricans* toad with standard or distress call vocalization). Vertical color lines indicate the estimated classification. Therefore, a pattern is correctly classified if its line color matches the band color.

As it can be seen, most of the correctly classified patterns have high Q values. Therefore, Q can be used to determine a confidence degree in the classification result.

**Figure 17:**
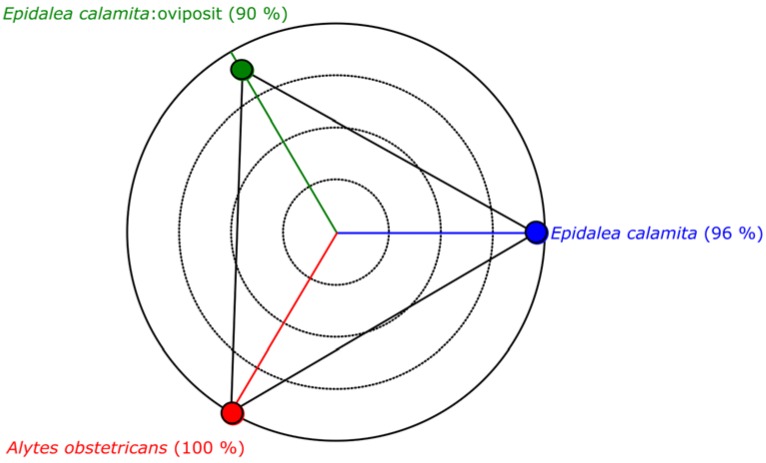
Performance of the modified maximum likelihood classifier.

Figure 17 depicts the performance of the modified maximum likelihood classifier, according to the results obtained with the audio collection. As it can be seen, this classifier offers a very good classification, with a success rate higher than 98%.

## 5. Conclusions and Future Work

This paper describes a classification architecture for animal voice recognition. The main goal of the proposed architecture is first to reduce biologists’ effort to manually classify audio patterns, and second, to provide a method that can be used to classify classes not previously considered. This classification architecture is based on the use of MPEG-7 low level descriptors, plus a family of derived descriptors used to detect animal voice reverberations. These descriptors have been tested in real scenarios detecting anuran voices stored in a public sound database.

The obtained results allow us to conclude that the proposed generic descriptors, using adequate classifiers, can be used to classify animal voices with a high degree of accuracy. In addition, the proposed architecture does not have the most common drawbacks of audio classification architectures.

The future work that is being considered by the authors focuses on two strategies: first, the deployment of this animal voice detection system in several natural environments; and secondly, the development of other classifiers using the proposed descriptors.

## Figures and Tables

**Table 1:** Analysis of the sound collections.

Caption	Vocalization	Files
*Epidalea calamita*	standard	20
	chorus	3
	oviposit	10
*Alytes obstetricans*	standard	29
	distress call	1
